# Substance Use Emergency Department Visits Among Youths With Chronic Conditions During COVID-19

**DOI:** 10.1001/jamanetworkopen.2024.35059

**Published:** 2024-10-04

**Authors:** Faith Summersett Williams, Isabella Zaniletti, Abbey R. Masonbrink, Robert Garofalo, Maria Rahmandar, Niranjan S. Karnik, Geri Donenberg, Lisa Kuhns

**Affiliations:** 1 Department of Pediatrics, Northwestern University, Feinberg School of Medicine, Ann & Robert H. Lurie Children’s Hospital, Chicago, Illinois; 2 Children’s Hospital Association, Lenexa, Kansas; 3 Department of Pediatrics, University of Missouri-Kansas City School of Medicine, Children’s Mercy Kansas City; 4 Institute for Juvenile Research, Department of Psychiatry, College of Medicine, University of Illinois at Chicago; 5 Department of Medicine, University of Illinois at Chicago, Center for Dissemination and Implementation Science

## Abstract

**Importance:**

Pediatric emergency department (ED) visits for substance use (SU) increased during COVID-19. Better understanding of trends associated with SU ED visits among youths with a chronic medical condition (CMC) is needed to target SU screening, prevention, and intervention efforts in this population.

**Objective:**

To describe trends in pediatric SU ED visits before and during COVID-19 among youths in the US with and without CMCs and by race and ethnicity.

**Design, Setting, and Participants:**

In this cohort study, data were obtained from 47 US children’s hospital EDs in the Pediatric Health Information System (PHIS) between March 1, 2018, and March 1, 2022. The cohort included patients aged 10 to 18 years. Data analysis occurred from November 2022 to February 2023.

**Exposures:**

ED visit occurrence before or during the COVID-19 pandemic and with or without the presence of a CMC. CMCs included chronic conditions (CCs) and complex CCs (CCCs).

**Main Outcomes and Measures:**

The primary outcome was the number of visits for an SU diagnosis based on diagnostic codes for youths with and without CMCs. Trends were assessed using logistic regression, adjusting for covariates and center effect.

**Results:**

The sample included 3 722 553 ED visits from March 1, 2018, to March 1, 2022 (1 932 258 aged 14-18 years [51.9%]; 1 969 718 female [52.9%]; 961 121 Hispanic [25.8%]; 977 097 non-Hispanic Black [26.2%]; 1 473 656 non-Hispanic White [39.6%]). Of all visits, 1 016 913 (27.3%) were youths with CCs and 367 934 (9.9%) were youths with CCCs. Youth SU ED visits increased for all groups during COVID-19. The SU ED visits increased by 23% for youths with CCs (21 357 visits [4.0%] to 23 606 visits [4.9%]), by 26% for youths with CCCs (3594 visits [1.9%] to 4244 visits [2.4%]), and by 50% for youths without CCs (4997 visits [0.4%] to 5607 visits [0.6%]). Furthermore, compared with youths without CCs, youths with CCs had consistently larger odds of SU than the other groups before COVID-19 (adjusted odds ratio, 9.74; 99% CI, 9.35-10.15) and during COVID-19 (adjusted odds ratio, 8.58; 99% CI, 8.25-8.92). The interaction between race and ethnicity and CMCs was significant (*P *for interaction < .001).

**Conclusions and Relevance:**

The findings of this cohort study suggest that providing SU services to all youths during times of societal crises is critical, but particularly for youths with CMCs who experience higher potential health impacts from SU given their medical concerns.

## Key Points

**Question:**

During the COVID-19 pandemic, did youths with chronic conditions or complex chronic conditions have more emergency department (ED) visits for substance use (SU) than youths without chronic conditions?

**Findings:**

This cohort study of 3 722 553 ED visits from 47 US children’s hospitals from March 2018 to March 2022 found that youth SU ED visits increased during the COVID-19 pandemic in comparison with the pre–COVID-19 period, with the largest increase seen in those with chronic conditions.

**Meaning:**

These findings of increased SU ED visits among youths with chronic conditions suggest that efforts are needed to improve hospital-based SU care for this population.

## Teaser

This cohort study analyzes changes in pediatric emergency department (ED) visits for substance use before and during the COVID-19 pandemic among youths with chronic medical conditions in the US.

## Introduction

Chronic medical conditions (CMCs) are broadly defined as conditions that last 1 year or longer, require ongoing medical attention, and/or limit activities of daily living.^1^ Youths with CMCs are equally or more likely to engage in substance use (SU), such as use of alcohol, cannabis, and nicotine, compared with youths without CMCs.^2,3^ Although similar proportions of youths with and without CMCs experiment with substances in early adolescence, youths with CMCs are more likely to engage in problematic alcohol and other SU and to receive a SU disorder diagnosis by older adolescence and young adulthood.^3,4^ SU in all youths poses risks for numerous adverse outcomes, including acute harm from accidents and injuries, negative impact on youth brain development, mental illness, and difficulties with educational and employment attainment.^5^ Importantly, SU in youths with CMCs poses additional risks including adverse medication interactions, increased treatment nonadherence, poor disease control, and higher likelihood of prescription medication misuse and accidental death.^4,6,7^

Adolescence is a developmental period characterized by rapid biological (eg, hormonal) and social (eg, peer acceptance) changes.^8^ The increased need for autonomy, exploration, and peer acceptance, especially among youths with medical complexities may contribute to SU, particularly when these youths are medically stable and engaged in academic and social activities with their peers. However, SU among youths with CMCs may uniquely undermine health status and disease management. SU in this population has negative implications for mental health outcomes, including higher likelihood of polysubstance use, addiction, anxiety, and/or depressive disorders in later adolescence and early adulthood, as well as medical outcomes associated with potential medication interactions, treatment nonadherence, poor disease management, and accidental death.^4,9^

The COVID-19 pandemic has given rise to concerns about the social well-being of youths, including its potential to increase or exacerbate SU among youths, especially individuals with CMCs.^10^ Current literature on youth self-reported SU during COVID-19 is mixed, with some studies finding increased alcohol and cannabis use, and others finding decreased electronic cigarette use or no significant change in cannabis use or binge drinking.^11,12,13,14^ However, during COVID-19, deaths due to drug overdose increased sharply, and more than doubled among youths and young adults.^15,16,17^

SU emergency department (ED) visits and hospitalizations among youths with CMC may be a more objective indicator of problematic SU compared with self-reported screeners because SU ED visits can offer the benefit of confirming SU via a urine drug screen and an assessment by a licensed clinician. A recent study^18^ of youth hospital visits for a SU diagnosis before COVID-19 (ie, 2017-2019) and during COVID-19 (2020) found increased SU during the initial summer of the COVID-19 pandemic (ie, June 2020 to August 2020) compared with the summer before COVID-19 (ie, June 2017 to August 2019), particularly among younger age groups, historically racialized Black and Hispanic youths, and those with underlying mental health disorders. This finding underscores the urgent need to support youths at risk of substance-related harm. However, research has not examined SU ED visits among youths with CMC before or during COVID-19.

Youths with CMCs are often categorized into 2 groups—youths with chronic conditions (CCs) and youths with complex CCs (CCCs). This categorization distinguishes youths with CCs affecting a single body system vs those who have any type of medical condition that can be expected to last more than 12 months (unless death intervenes) and involves several different organ systems that require specialty pediatric care and/or hospitalization in a tertiary care center (ie, CCCs^19^). Highlighting this distinction among youths with CMCs is important because youths with CCCs have more intensive treatment plans that often result in longer hospitalizations compared with those with CCs.^20^ Moreover, clinicians caring for youths with CCCs often find it difficult to determine discharge readiness given that many youths with CCCs do not return to a completely healthy baseline compared with youths with CCs.^20^ As a result, identifying differences in the prevalence rates of SU between these groups can inform patient, clinician, and organizational-level efforts to appropriately address SU in this population, particularly during crisis events, such as the COVID-19 pandemic.

We aimed to address this gap in the published literature by examining trends in SU diagnoses among youths with CMCs (those with CCs and CCCs) using health systems data from a national sample of children’s hospital EDs before and during COVID-19. We assessed differences in patient and clinical characteristics. Given prior findings, we hypothesized that SU ED visits would increase during the COVID-19 pandemic, compared with the prior period, and youths with CMCs would have a greater probability of SU ED visits than those without CMCs during the COVID-19 pandemic.

## Methods

This cohort study was deemed exempt from review and the requirement of informed consent by the institutional review board at Ann & Robert H. Lurie Children’s Hospital of Chicago. The study followed the Strengthening the Reporting of Observational Studies in Epidemiology (STROBE) reporting guideline.

### Study Design and Participants

We conducted a retrospective analysis of hospital ED encounters among patients aged 10 to 18 years in the Pediatric Health Information System (PHIS; Children’s Hospital Association, Lenexa, Kansas) database from March 2018 through March 2022. PHIS contains up to 41 diagnoses on each encounter using the *International Statistical Classification of Diseases, Tenth Revision, Clinical Modification (ICD-10-CM)*.^21^ Hospitals across the US that provided data throughout the study period were included (47 out of 49 children’s hospitals). Due to different mitigation strategies during COVID-19, we defined the comparison periods as before COVID-19 (March 1, 2018, to February 28, 2022) and during COVID-19 (March 1, 2020, to February 28, 2022).

The primary outcome was the number of youth visits with a SU diagnosis (primary or secondary diagnosis), including alcohol, opioid, cannabis, nicotine, sedative, cocaine, hallucinogen, inhalant, other stimulant, and other psychoactive SU, before COVID-19 compared with during COVID-19, defined by *ICD-10-CM* codes (eTable in Supplement 1).

The primary independent variable was CC status. To facilitate comparison of SU ED visits by CC status, we used a hierarchical approach to categorize youths into 3 groups based on their medical diagnosis: (1) CCC; 2) CC, measured by the CC indicator; and (3) no CC or CCC. We used the Feudtner CCC classification system, an algorithm that classifies patients into categories based on their *International Statistical Classification of Diseases and Related Health Problems, Tenth Revision (ICD-10) *diagnosis and procedure codes (eg, cardiovascular, respiratory, neuromuscular, kidney, gastrointestinal, hematologic or immunologic, metabolic, other congenital or genetic, malignant neoplasm, and premature and neonatal category with codes for medical devices and transplantation for most categories^22^). The CC indicator was defined using the Agency for Healthcare Research and Quality definition of patients with CCs such as malignant cancer, diabetes, obesity, hypertension, and most mental health conditions, based on their *ICD-10-CM* diagnosis codes.^23^

### Statistical Analysis

We summarized categorical data as counts and percentages, and continuous variables as median and IQR. We compared group frequencies using χ^2^ tests and used the Wilcoxon rank sum test and Kruskal-Wallis test for continuous distributions. We modeled the binary outcome of SU using logistic regression, adjusting the CC group odds for covariates. To evaluate whether the CC groups differed before vs during the COVID-19 pandemic, we evaluated the interaction of CC group by time. Covariates included age, sex, race and ethnicity, payer type, Childhood Opportunity Index (COI; a measure of the quality of neighborhood resources and conditions that contribute to healthy child development),^24^ and hospital. Race and ethnicity were obtained via electronic health records and included Hispanic, non-Hispanic Asian, non-Hispanic Black, non-Hispanic White, and other (defined as multiracial, American Indian and Alaksa Native, and Pacific Islander). We evaluated the interaction of CC group by race and ethnicity interaction because there are known disparities in youth SU patterns and treatment among minoritized racial and ethnic groups.^25,26^ Covariates included age, sex, race and ethnicity, payer type, COI, and hospital. We reported the adjusted odds ratios (aORs) and their 99% CIs, and we graphically displayed the percentage of SU before and after the COVID-19 pandemic. We used listwise deletion to handle missing data. All analyses were conducted in SAS Enterprise Guide version 8.3 (SAS Institute) at a significance level of *P* < .01. Data analysis occurred from November 2022 to February 2023.

## Results

This sample included 3 722 553 ED visits from March 1, 2018, to March 1, 2022 (1 932 258 aged 14-18 years [51.9%]; 1 969 718 female [52.9%]; 961 121 Hispanic [25.8%]; 977 097 non-Hispanic Black [26.2%]; 1 473 656 non-Hispanic White [39.6%]) (Table 1). A total of 2 067 382 visits were included from the pre–COVID-19 time period (March 1, 2018, to February 29, 2020) and 1 655 171 visits were included from the during–COVID-19 time period (March 1, 2020, to March 1, 2022). Across all time periods, youths with a very low COI accounted for 1 080 691 visits (29.0%), and youths from the Southern region of the US accounted for 1 435 319 visits (38.6%). Furthermore, 2 337 706 visits during the study period (62.8%) were youths without CCs, 1 016 913 visits (27.3%) were youths with CCs, and 367 934 (9.9%) were youths with CCCs. Youth SU ED visits (for any substance) significantly increased overall from before COVID-19 (29 948 visits [1.4%]) to during COVID-19 (33 457 visits [2.0%) (*P* < .001) (Table 1). All individual SU diagnoses, except those associated with alcohol, cocaine, and other stimulant, increased during COVID-19 compared with before COVID-19 (Table 1).

**Table 1.** Patient Characteristics by Time Period

Characteristic	Participants, No. (%)			*P* value
	Overall (N = 3 722 553)	Before COVID-19 (n = 2 067 382)	During COVID-19 (n = 1 655 171)	
Race and ethnicity				
Hispanic	961 121 (25.8)	523 831 (25.3)	437 290 (26.4)	
Non-Hispanic Asian	73 087 (2.0)	39 927 (1.9)	33 160 (2.0)	<.001
Non-Hispanic Black	977 097 (26.2)	541 682 (26.2)	435 415 (26.3)	
Non-Hispanic other^a^	237 592 (6.4)	151 929 (7.3)	85 663 (5.2)	
Non-Hispanic White	1 473 656 (39.6)	810 013 (39.2)	663 643 (40.1)	
Age, y				
10-13	1 790 295 (48.1)	1 036 379 (50.1)	753 916 (45.5)	<.001
14-18	1 932 258 (51.9)	1 031 003 (49.9)	901 255 (54.5)	
Childhood Opportunity Index				
Very low	1 080 691 (29.0)	618 401 (29.9)	462 290 (27.9)	<.001
Low	691 818 (18.6)	386 426 (18.7)	305 392 (18.5)	
Moderate	629 895 (16.9)	344 300 (16.7)	285 595 (17.3)	
High	586 538 (15.8)	317 582 (15.4)	268 956 (16.2)	
Very high	723 998 (19.4)	393 091 (19.0)	330 907 (20.0)	
Missing	9613 (0.3)	7582 (0.4)	2031 (0.1)	
Sex				
Male	1 751 878 (47.1)	985 198 (47.7)	766 680 (46.3)	<.001
Female	1 969 718 (52.9)	1 081 703 (52.3)	888 015 (53.7)	
Insurance type				
Government	2 104 074 (56.5)	1 184 197 (57.3)	919 877 (55.6)	<.001
Private	1 488 965 (40.0)	827 562 (40.0)	661 403 (40.0)	
Other	129 514 (3.5)	55 623 (2.7)	73891 (4.5)	
Region				
Midwest	1 154 375 (31.0)	659 654 (31.9)	494 721 (29.9)	<.001
Northeast	409 300 (11.0)	236 684 (11.4)	172 616 (10.4)	
South	1 435 319 (38.6)	754 006 (36.5)	681 313 (41.2)	
West	723 559 (19.4)	417 038 (20.2)	306 521 (18.5)	
Urban vs rural	3 489 256 (93.7)	1 942 666 (94.0)	1 546 590 (93.4)	<.001
Substance use diagnosis				
Any	63 405 (1.7)	29 948 (1.4)	33 457 (2.0)	<.001
Alcohol	10 099 (0.3)	5071 (0.2)	5028 (0.3)	<.001
Opioid	2663 (0.1)	1168 (0.1)	1495 (0.1)	<.001
Cannabis	36 780 (1.0)	16 753 (0.8)	20 027 (1.2)	<.001
Sedative	3088 (0.1)	1480 (0.1)	1608 (0.1)	<.001
Cocaine	1152 (<0.0)	644 (<0.0)	508 (<0.0)	.80
Other stimulant	1838 (<0.0)	987 (<0.0)	851 (0.1)	.11
Hallucinogen	805 (<0.0)	363 (<0.0)	442 (<0.0)	<.001
Nicotine	14 006 (0.4)	6968 (0.3)	7038 (0.4)	<.001
Inhalant	162 (<0.0)	78 (<0.0)	84 (<0.0)	.06
Other psychoactive	6090 (0.2)	2849 (0.1)	3241 (0.2)	<.001
Chronic condition group				
None	2 337 706 (62.8)	1 335 370 (64.6)	1 002 336 (60.6)	<.001
Chronic condition	1 016 913 (27.3)	538 448 (26.0)	478 465 (28.9)	
Complex chronic condition	367 934 (9.9)	193 564 (9.4)	174 370 (10.5)	
Complex chronic condition type				
Neuromuscular	80 085 (2.2)	42 792 (2.1)	37 293 (2.3)	<.001
Cardiovascular	65 833 (1.8)	33 162 (1.6)	32 671 (2.0)	<.001
Respiratory	16 506 (0.4)	9358 (0.5)	7148 (0.4)	.003
Kidney	32 310 (0.9)	16 815 (0.8)	15 495 (0.9)	<.001
Gastrointestinal	93 093 (2.5)	47 692 (2.3)	45 401 (2.7)	<.001
Hematologic or immunologic	67 886 (1.8)	35 503 (1.7)	32 383 (2.0)	<.001
Metabolic	75 422 (2.0)	36 940 (1.8)	38 482 (2.3)	<.001
Genetic	41 613 (1.1)	22 933 (1.1)	18 680 (1.1)	.08
Malignant neoplasm	37 456 (1.0)	20 328 (1.0)	17 128 (1.0)	<.001
Neonatal	1983 (0.1)	1082 (0.1)	901 (0.1)	.38
Transplant	1948 (0.1)	1087 (0.1)	861 (0.1)	.81
Technology dependent	91 259 (2.5)	49 545 (2.4)	41 714 (2.5)	<.001
Disposition				
Home health	10 253 (0.3)	6744 (0.3)	3509 (0.2)	<.001
Home	3 552 795 (95.4)	1 985 246 (96.0)	1 567 549 (94.7)	
Other	137 877 (3.7)	64 650 (3.1)	73 227 (4.4)	
Skilled facility	21 628 (0.6)	10 742 (0.5)	10 886 (0.7)	
Mortality	2362 (0.1)	1073 (0.1)	1289 (0.1)	<.001
Length of stay, d				
Median (IQR)	1 (1-1)	1 (1-1)	1 (1-1)	<.001
Mean (SD)	1.5 (3.5)	1.4 (3.2)	1.6 (3.8)	

^a^ Other was defined as multiracial, American Indian and Alaska Native, and Pacific Islander.

Table 2 displays demographic characteristics and type of SU ED visit by CC group at all time periods. Across all SU diagnosis types, there were more SU ED visits among youths with CCs (44 963 visits [4.4%]) and CCCs (7838 visits [2.1%]) compared with youths without CCs (10 604 visits [0.5%]) (*P* < .001). By substance type, the most frequent SU diagnoses in ED visits among youths were cannabis (CCs, 24 670 cases [2.4%]; CCCs, 4709 cases [1.3%]; no CC, 7401 cases [0.3%]; *P* < .001), nicotine (CCs, 12 274 cases [1.2%]; CCCs, 1731 cases [0.5%]; no CC, 1 case [<0.0%]; *P* < .001), alcohol (CCs, 7223 cases [0.7%]; CCCs, 883 cases [0.2%]; no CC, 1993 cases [0.1%]; *P* < .001), and opioid (CCs, 1612 cases [0.2%]; CCCs, 704 cases [0.2%]; no CC, 347 cases [<0.0%]; *P* < .001) (Table 2).

**Table 2.** Patient Characteristics by Chronic Condition Group^a^^,^^b^

Characteristic	Participants, No. (%)				*P* value
	Overall (N = 3 722 553)	None (n = 2 337 706)	Chronic condition (n = 1 016 913)	Complex chronic condition (n = 367 934)	
Race and ethnicity					
Hispanic	961 121 (25.8)	652 234 (27.9)	227 706 (22.4)	81 181 (22.1)	<.001
Non-Hispanic Asian	73 087 (2.0)	48 063 (2.1)	16 483 (1.6)	8541 (2.3)	
Non-Hispanic Black	977 097 (26.2)	593 269 (25.4)	284 931 (28.0)	98 897 (26.9)	
Non-Hispanic other^c^	237 592 (6.4)	155 344 (6.6)	62 174 (6.1)	20 074 (5.5)	
Non-Hispanic White	1 473 656 (39.6)	888 796 (38)	425 619 (41.9)	159 241 (43.3)	
Age, y					
10-13	1 790 295 (48.1)	1 196 113 (51.2)	444 592 (43.7)	149 590 (40.7)	<.001
14-18	1 932 258 (51.9)	1 141 593 (48.8)	572 321 (56.3)	218 344 (59.3)	
Childhood Opportunity Index					
Very low	1 080 691 (29.0)	701 243 (30.0)	289 042 (28.4)	90 406 (24.6)	<.001
Low	691 818 (18.6)	435 572 (18.6)	184 508 (18.1)	71 738 (19.5)	
Moderate	629 895 (16.9)	385 656 (16.5)	175 257 (17.2)	68 982 (18.7)	
High	586 538 (15.8)	355 923 (15.2)	165 904 (16.3)	64 711 (17.6)	
Very high	723 998 (19.4)	452 792 (19.4)	200 206 (19.7)	71 000 (19.3)	
Missing	9613 (0.3)	6520 (0.3)	1996 (0.2)	1097 (0.3)	
Sex					
Male	1 751 878 (47.1)	1 124 325 (48.1)	447 618 (44.0)	179 935 (48.9)	<.001
Female	1 969 718 (52.9)	1 212 782 (51.9)	569 012 (56.0)	187 924 (51.1)	
Insurance type					
Government	2 104 074 (56.5)	1 311 766 (56.1)	581 550 (57.2)	210 758 (57.3)	<.001
Private	1 488 965 (40.0)	944 652 (40.4)	400 367 (39.4)	143 946 (39.1)	
Other	129 514 (3.5)	81 288 (3.5)	34 996 (3.4)	13 230 (3.6)	
Region					
Midwest	1 154 375 (31.0)	800 166 (34.2)	267 885 (26.3)	86 324 (23.5)	<.001
Northeast	409 300 (11.0)	221 982 (9.5)	138 648 (13.6)	48 670 (13.2)	
South	1 435 319 (38.6)	867 109 (37.1)	407 290 (40.1)	160 920 (43.7)	
West	723 559 (19.4)	448 449 (19.2)	203 090 (20.0)	72 020 (19.6)	
Urban vs rural	3 489 256 (93.7)	2 212 769 (94.7)	948 152 (93.2)	328 335 (89.2)	<.001
Substance use diagnosis					
Any	63 405 (1.7)	10 604 (0.5)	44 963 (4.4)	7838 (2.1)	<.001
Alcohol	10 099 (0.3)	1993 (0.1)	7223 (0.7)	883 (0.2)	<.001
Opioid	2663 (0.1)	347 (<0.0)	1612 (0.2)	704 (0.2)	<.001
Cannabis	36 780 (1.0)	7401 (0.3)	24 670 (2.4)	4709 (1.3)	<.001
Sedative	3088 (0.1)	255 (<0.0)	2313 (0.2)	520 (0.1)	<.001
Cocaine	1152 (<0.0)	109 (<0.0)	888 (0.1)	155 (<0.0)	<.001
Other stimulant	1838 (<0.0)	178 (<0.0)	1448 (0.1)	212 (0.1)	<.001
Hallucinogen	805 (<0.0)	93 (<0.0)	638 (0.1)	74 (<0.0)	<.001
Nicotine	14 006 (0.4)	1 (<0.0)	12 274 (1.2)	1731 (0.5)	<.001
Inhalant	162 (<0.0)	25 (<0.0)	121 (<0.0)	16 (<0.0)	<.001
Other psychoactive	6090 (0.2)	747 (<0.0)	4797 (0.5)	546 (0.1)	<.001
Disposition					
Home health	10 253 (0.3)	1972 (0.1)	2065 (0.2)	6216 (1.7)	<.001
Home	3 552 795 (95.4)	2 278 284 (97.5)	930 525 (91.5)	343 986 (93.5)	
Other	137 877 (3.7)	49 290 (2.1)	73 741 (7.3)	14 846 (4.0)	
Skilled facility	21 628 (0.6)	8160 (0.3)	10 582 (1.0)	2886 (0.8)	
Mortality	2362 (0.1)	97 (<0.0)	434 (<0.0)	1831 (0.5)	<.001
Length of stay, d					
Median (IQR)	1 (1-1)	1 (1-1)	1 (1-1)	1 (1-3)	<.001
Mean (SD)	1.5 (3.5)	1.1 (0.6)	1.7 (3.0)	3.8 (9.4)	

^a^ χ^2^ tests were used for categorical variables (summarized as count and percentages).

^b^ Kruskal-Wallis (3-group) and Wilcoxon rank sum test (2-group) were used for continuous variables (summarized as median and IQR).

^c^ Other was defined as multiracial, Native American, and Pacific Islander.

**Figure 1.**
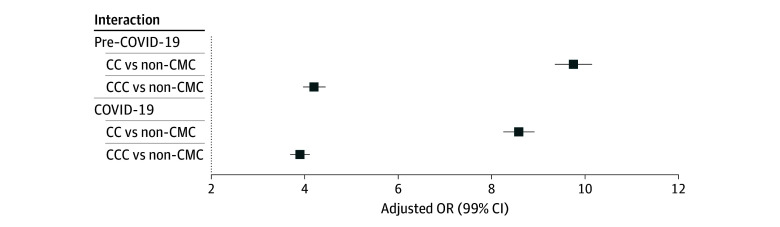
Odds of Substance Use Visits by Chronic Medical Condition (CMC) Group The reference group for each interaction was non-CMC. CC indicates chronic condition; CCC, complex chronic condition; OR, odds ratio.

The number of SU ED visits increased for all groups during COVID-19 (eFigure in Supplement 1). The SU ED visits increased by 23% for youths with CCs (21 357 visits [4.0%] to 23 606 visits [4.9%]), by 26% for youths with CCCs (3594 visits [1.9%] to 4244 visits [2.4%]), and by 50% for youths without CCs (4997 visits [0.4%] to 5607 visits [0.6%]). Although the gap in the odds of SU between the youths with CCs and CCCs compared with the youths without CCs reduced during COVID-19 (*P *for interaction < .001), youths with CCs had consistently larger odds of SU than the other groups before COVID-19 (aOR, 9.74; 99% CI, 9.35-10.15) and during COVID-19 (aOR, 8.58; 99% CI, 8.25-8.92) (Figure 1 and Table 3).

**Table 3.** Interactions Between CMC and Time Period and CMC and Race and Ethnicity

Characteristic	Adjusted OR (99% CI)^a^	*P* value
CC group		
None	1 [Reference]	NA
CC	11.94 (11.30-12.61)	<.001
CCC	5.10 (4.74-5.48)	
Time period		
Before COVID-19	1 [Reference]	NA
During COVID-19	1.35 (1.28-1.42)	<.001
CC × time period interaction		
Pre–COVID-19		
CC vs non-CMC	9.74 (9.35-10.15)	<.001
CCC vs non-CMC	4.20 (3.97-4.45)	
During COVID-19		
CC vs non-CMC	8.58 (8.25-8.92)	<.001
CCC vs non-CMC	3.90 (3.69-4.11)	
Race and ethnicity		
Hispanic	1.08 (1.01-1.15)	<.001
Non-Hispanic Asian	1.26 (1.06-1.50)	
Non-Hispanic Black	1.08 (1.01-1.15)	
Non-Hispanic other^b^	1.21 (1.09-1.34)	
Non-Hispanic White	1 [Reference]	NA
CC × race and ethnicity interaction		
CCC		
Non-Hispanic Black vs non-Hispanic White	0.81 (0.75-0.87)	<.001
Hispanic vs non-Hispanic White	0.71 (0.65-0.78)	
Asian vs non-Hispanic White	0.45 (0.34-0.60)	
Non-Hispanic other vs non-Hispanic White^b^	0.96 (0.84-1.09)	
CC		
Non-Hispanic Black vs non-Hispanic White	0.71 (0.68-0.73)	<.001
Hispanic vs non-Hispanic White	0.80 (0.77-0.83)	
Non-Hispanic Asian vs non-Hispanic White	0.57 (0.50-0.65)	
Non-Hispanic other vs non-Hispanic White^b^	0.93 (0.88-0.99)	
Non-CMC		
Non-Hispanic Black vs non-Hispanic White	1.04 (0.97-1.11)	<.001
Hispanic vs non-Hispanic White	1.08 (1.01-1.15)	
Non-Hispanic Asian vs non-Hispanic White	1.26 (1.06-1.50)	
Non-Hispanic other vs non-Hispanic White^b^	1.21 (1.09-1.34)	
Age, y		
10-13	1 [Reference]	NA
14-18	8.65 (8.34-8.97)	<.001
Childhood Opportunity Index		
Very low	1 [Reference]	NA
Low	1.03 (1.01-1.07)	<.001
Moderate	0.96 (0.93-1.00)	
High	0.99 (0.96-1.00)	
Very high	0.99 (0.96-1.03)	
Sex		
Male	1 [Reference]	NA
Female	0.74 (0.73-0.76)	<.001
Insurance type		
Government	1 [Reference]	NA
Private	0.87 (0.85-0.89)	<.001
Other	1.03 (0.97-1.10)	
Center, range	0.39-3.60	<.001

Abbreviations: CC, chronic condition; CCC, complex chronic condition; CMC, chronic medical condition; NA, not applicable; OR, odds ratio.

^a^ Adjusted for age, sex, race and ethnicity, payer type, Childhood Opportunity Index, and hospital.

^b^ Other was defined as defined as multiracial, Native American, and Pacific Islander.

**Figure 2.**
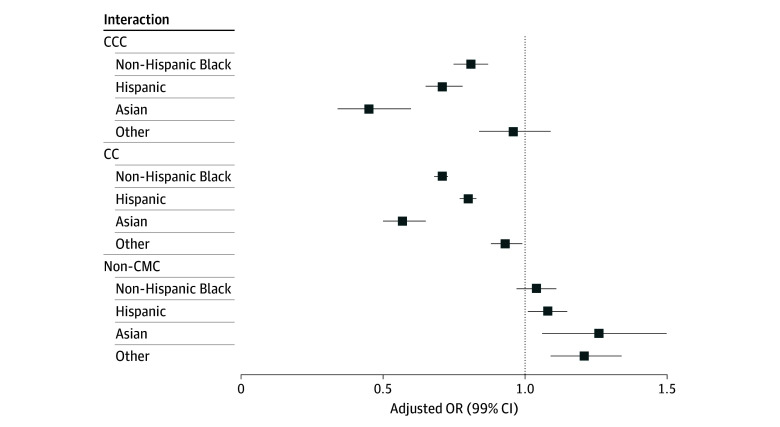
Odds of Substance Use by Chronic Medical Condition (CMC) Group and Race and Ethnicity The reference for each group condition category was non-Hispanic White. Other was defined as multiracial, Native American, and Pacific Islander. CC indicates chronic condition; CCC, complex chronic condition; OR, odds ratio.

The interaction between race and ethnicity and CMCs was significant (*P *for interaction < .001). Compared with non-Hispanic White youths in the non-CMC group, there were higher odds of SU ED visits for non-Hispanic Asian youths (aOR, 1.26; 99% CI, 1.06-1.50) and Hispanic youths (aOR, 1.08; 99% CI, 1.01-1.15) in the non-CMC group. Conversely, compared with non-Hispanic White youths in both the CC and CCC groups, there were lower odds of SU ED visits for non-Hispanic Asian youths (aOR for CC, 0.57; 99% CI, 0.50-0.65; aOR for CCC, 0.45; 99% CI, 0.34-0.60), non-Hispanic Black youths (aOR for CC, 0.71; 99% CI, 0.68-0.73; aOR for CCC, 0.81; 99% CI, 0.75-0.87), and Hispanic youths (aOR for CC, 0.80; 99% CI, 0.77-0.83; aOR for CCC, 0.71; 99% CI, 0.65-0.78) (Figure 2 and Table 3).

## Discussion

In this cohort study, we found an interaction between CMC and the pre–COVID-19 and during–COVID-19 time periods; youths with CCs and youths with CCCs had significantly higher odds of a SU ED visit compared with youths without CCs. Specifically, youths with CCs had 10 times more SU ED visits, and youths with CCCs had 4 times more SU ED visits than youths with no CCs or CCCs before COVID-19. During COVID-19, youths with CCs had 9 times more SU ED visits, and youths with CCCs had 4 times more SU ED visits than youths with no CCs or CCCs. The interaction between SU and time indicates that youths may have experienced increased challenges during COVID-19 as a result of public health efforts (eg, social isolation and school closures) to decrease disease transmission. Recent studies^27,28^ have found that social distancing measures and virtual learning were associated with an increase in the occurrence of mental health symptoms, which likely influenced an increase in SU. Furthermore, over all periods, racially and ethnically minoritized youths with CCs or CCCs had higher odds of SU ED visits compared with non-Hispanic White youths with CCs and CCCs. These findings may inform future efforts to mitigate risk related to SU among this particularly vulnerable pediatric population.

Our findings have important implications for clinical practice and highlight the need for universal SU screening and intervention in pediatric ED settings and potentially other pediatric health care settings where youths, particularly those with CMCs, may seek care. Many youths do not access primary care, nor do they receive preventive counseling (eg, on SU or sexual and reproductive health) when they do attend primary care visits^29,30^; thus, our data suggest that nontraditional settings, such as ED encounters, represent an important and potentially underutilized opportunity to address SU in this population. Among youths with CMCs, pediatric hospital or subspecialty clinicians who treat youths with CMCs may underestimate risk for SU, may assume SU screening and counseling is occurring in primary care settings, or may not prioritize addressing SU in this population given the complexities involved in the management of their medical conditions. These assumptions may result in missed opportunities for early SU intervention and fragmented care that negatively impacts both overall health and the effective delivery of SU services when warranted. To our knowledge, most pediatric hospitals have not implemented universal SU screening, brief intervention, or referral resources either in the ED or in other clinical settings where youths with CMCs may be likely to access care (eg, inpatient units).^31,32^ Known barriers for implementing targeted or universal SU screening as well as potential treatment and/or referral for services include clinicians underestimating the scope of and risks associated with SU among medically vulnerable youths, limited clinician training and knowledge on evidence-based SU care for youths, stigma attached to SU, confidentiality concerns, and limited mental and behavioral health resources.^33,34,35,36^ Efforts to increase SU care for youths should focus on clinician training in evidence-based SU treatment, development of specific SU resources such as methods for confidential screening and treatment, and support for mental and behavioral health resources in this setting.^37,38^

Further work is needed to improve SU screening and treatment specifically for adolescents with CCs and CCCs because of potential underlying SU-related risk factors and comorbid conditions. For example, adolescents with CCs and CCCs may be engaging in SU as a form of self-medication for medical (eg, chronic pain) and mental health (eg, depression, anxiety, or posttraumatic stress) symptoms.^39,40,41^ Thus, brief SU interventions offered by hospital-based clinicians (eg, physicians, nurses, and social workers) should incorporate specific resources to screen for these potential mental and physical health comorbidities and include tailored condition-specific counseling and education. These SU interventions should also address the complexity of navigating disease management and youth mental health for this medically vulnerable population. Furthermore, the application of implementation science methods is needed to identify strategies that will facilitate the integration of universal SU screening and counseling in pediatric health systems.

We found that racially and ethnically minoritized youths with CCs and CCCs had lower odds of SU ED visits compared with non-Hispanic White youths with CCs and CCCs. This finding could be in part explained by the fact that non-Hispanic White youths were potentially overrepresented in our data. On the other hand, this may be a conservative representation of SU among Black and Hispanic youths with CCs and CCCs given societal responses among the groups are typically different (eg, medicalization for White youths vs criminalization for racialized Black and Hispanic youths). Consistent with past literature, this finding may be driven by disparities in SU treatment for racialized youths,^42^ with systemic factors potentially contributing to disparities in SU treatment. These disparities further stress the importance of mainstreaming SU screening, brief intervention, and referral to treatment to understand racial and ethnic disparities in SU screening and treatment for youths with CMCs.

### Limitations

These findings should be viewed in light of several limitations. As with any retrospective database, use of administrative diagnosis codes may lead to misclassification due to coding error and/or reporting bias. Additionally, this study was limited to 47 hospitals that consistently provided data to PHIS between March 2018 and March 2022 and may not be representative of national pediatric or community hospital SU ED visit trends or trends in less resourced hospitals without the time and personnel to enter the data. Notably, longitudinal analyses of claims data are aggregates and not person level trajectories. Furthermore, we did not account for hospital-level variation in our trend analysis; as such, hospital-specific trends may differ from the national estimate. Moreover, child-, parent-, and community-level variables were not available to assess social determinants of health related to COVID-19 (eg, parental unemployment or loss of health insurance) that may disproportionately impact SU ED visits.

## Conclusions

Our cohort study offers novel information and reveals concerning trends of SU ED visits during the COVID-19 pandemic (compared with pr–COVID-19), particularly in youths with CCs and CCCs. Future efforts are needed to improve SU screening and treatment as the standard of care for this population. Further research to understand system- and patient-level factors, including racial and ethnic disparities, can inform efforts to improve hospital-based SU care and offset risk for numerous adverse outcomes related to SU in youths with CMCs.

## Notes

Supplement 1. **eTable.** Substance Use *ICD-10* Codes **eFigure.** Percentage of CMC Groups by Time Period

Supplement 2.
Data Sharing Statement
