# Supplementary material for: Substance Use Emergency Department Visits Among Youths With Chronic Conditions During COVID-19
Source: JAMA Netw Open. 2024 Oct 4;7(10):e2435059. doi: 10.1001/jamanetworkopen.2024.35059 (PMC11581538; doi:10.1001/jamanetworkopen.2024.35059)
Supplement: Supplement 1. — eTable. Substance Use ICD-10 Codes eFigure. Percentage of CMC Groups by Time Period [file jamanetwopen-e2435059-s001.pdf]

Supplemental Online Content

Williams FS, Zaniletti I, Masonbrink AR, et al. Substance use emergency department visits among youths with chronic conditions during COVID-19. *JAMA Netw. Open*. 2024;7(9):e2435059. doi:10.1001/jamanetworkopen.2024.35059

eTable. Substance Use *ICD-10* Codes

eFigure. Percentage of CMC Groups by Time Period

This supplemental material has been provided by the authors to give readers additional information about their work.

© 2024 Williams FS et al. *JAMA Network Open.*

eTable. Substance Use *ICD-10* Codes

**Alcohol**							
'F10130',	'F10131',	'F10132',	'F10139',	'F10930',	'F10931',	'F10932',	'F10939',
'7903',	'9800',	'76071',	'O354XX0',	'O354XX1',	'O354XX2',	'O354XX3',	'O354XX4',
'O354XX5',	'O354XX9',	'P043',	'Q860',	'R780',	'O99310',	'O99315',	'G312',
'G621',	'I426',	'K2920',	'K2921',	'K700',	'K7010',	'K7011',	'K702',
'K7030',	'K7031',	'K7040',	'K7041',	'K709',	'O99311',	'O99312',	'O99313',
'O99314',	'F1010',	'F10120',	'F10121',	'F10129',	'F1014',	'F10150',	'F10151',
'F10159',	'F10180',	'F10181',	'F10182',	'F10188',	'F1019',	'F1020',	'F1021',
'F10220',	'F10221',	'F10229',	'F10230',	'F10231',	'F10232',	'F10239',	'F1024',
'F10250',	'F10251',	'F10259',	'F1026',	'F1027',	'F10280',	'F10281',	'F10282',
'F10288',	'F1029',	'F10920',	'F10921',	'F10929',	'F1094',	'F10950',	'F10951',
'F10959',	'F1096',	'F1097',	'F10980',	'F10981',	'F10982',	'F10988',	'F1099',
'T518X2A',	'T518X2S',	'T5192XA',	'T5192XS'				
**Opioid**							
'F1113',	'T402X1A',	'T402X4A',	'F1110',	'F11120',	'F11121',	'F11122',	'F11129',
'F1114',	'F11150',	'F11151',	'F11159',	'F11181',	'F11182',	'F11188',	'F1119',
'F1120',	'F1121',	'F11220',	'F11221',	'F11222',	'F11229',	'F1123',	'F1124',
'F11250',	'F11251',	'F11259',	'F11281',	'F11282',	'F11288',	'F1129',	'F1190',
'F11920',	'F11921',	'F11922',	'F11929',	'F1193',	'F1194',	'F11950',	'F11951',
'F11959',	'F11981',	'F11982',	'F11988',	'F1199',	'T398X2A',	'T398X2S',	'T3992XA',
'T3992XS',	'T402X2A',	'T402X2S',	'T507X2A',	'T507X2S'			
**Cannabis**							
'F1213',	'T407X1A',	'T407X4A',	'P0481',	'F1210',	'F12120',	'F12121',	'F12122',
'F12129',	'F12150',	'F12151',	'F12159',	'F12180',	'F12188',	'F1219',	'F1220',
'F1221',	'F12220',	'F12221',	'F12222',	'F12229',	'F1223',	'F12250',	'F12251',
'F12259',	'F12280',	'F12288',	'F1229',	'F1290',	'F12920',	'F12921',	'F12922',
'F12929',	'F1293',	'F12950',	'F12951',	'F12959',	'F12980',	'F12988',	'F1299',
'T407X2A',	'T407X2S'						
**Sedative**							
'F13130',	'F13131',	'F13132',	'F13139',	'T426X4A',	'P0417',	'F1310',	'F13120',
'F13121',	'F13129',	'F1314',	'F13150',	'F13151',	'F13159',	'F13180',	'F13181',

© 2024 Williams FS et al. *JAMA Network Open.*

	'F13182',	'F13188',	'F1319',	'F1320',	'F1321',	'F13220',	'F13221',	'F13229',
	'F13230',	'F13231',	'F13232',	'F13239',	'F1324',	'F13250',	'F13251',	'F13259',
	'F1326',	'F1327',	'F13280',	'F13281',	'F13282',	'F13288',	'F1329',	'F1390',
	'F13920',	'F13921',	'F13929',	'F13930',	'F13931',	'F13932',	'F13939',	'F1394',
	'F13950',	'F13951',	'F13959',	'F1396',	'F1397',	'F13980',	'F13981',	'F13982',
	'F13988',	'F1399',	'T426X2A',	'T426X2S',	'T4272XA',	'T4272XS'		
**Cocaine**								
	'F1413',	'F1493',	'97081',	'T405X1A',	'T405X4A',	'76075',	'P0441',	'R782', '30420',
	'30421',	'30422',	'30423',	'30560',	'30561',	'30562',	'30563',	'F1410',
	'F14120',	'F14121',	'F14122',	'F14129',	'F1414',	'F14150',	'F14151',	'F14159',
	'F14180',	'F14181',	'F14182',	'F14188',	'F1419',	'F1420',	'F1421',	'F14220',
	'F14221',	'F14222',	'F14229',	'F1423',	'F1424',	'F14250',	'F14251',	'F14259',
	'F14280',	'F14281',	'F14282',	'F14288',	'F1429',	'F1490',	'F14920',	'F14921',
	'F14922',	'F14929',	'F1494',	'F14950',	'F14951',	'F14959',	'F14980',	'F14981',
	'F14982',	'F14988',	'F1499',	'T405X2A',	'T405X2S'			
**Other Stimulant**								
	'F1513',	'F1510',	'F15120',	'F15121',	'F15122',	'F15129',	'F1514',	'F15150',
	'F15151',	'F15159',	'F15180',	'F15181',	'F15182',	'F15188',	'F1519',	'F1520',
	'F1521',	'F15220',	'F15221',	'F15222',	'F15229',	'F1523',	'F1524',	'F15250',
	'F15251',	'F15259',	'F15280',	'F15281',	'F15282',	'F15288',	'F1529',	'F1590',
	'F15920',	'F15921',	'F15922',	'F15929',	'F1593',	'F1594',	'F15950',	'F15951',
	'F15959',	'F15980',	'F15981',	'F15982',	'F15988',	'F1599'		
**Hallucinogen**								
	'T40901A',	'T40904A',	'T40991A',	'T40994A',	'P0442',	'R783',	'F1610',	'F16120',
	'F16121',	'F16122',	'F16129',	'F1614',	'F16150',	'F16151',	'F16159',	'F16180',
	'F16183',	'F16188',	'F1619',	'F1620',	'F1621',	'F16220',	'F16221',	'F16229',
	'F1624',	'F16250',	'F16251',	'F16259',	'F16280',	'F16283',	'F16288',	'F1629',
	'F1690',	'F16920',	'F16921',	'F16929',	'F1694',	'F16950',	'F16951',	'F16959',
	'F16980',	'F16983',	'F16988',	'F1699',	'T40902A',	'T40902S',	'T40992A',	'T40992S'
**Nicotine**								
	'F17200',	'F17201',	'F17203',	'F17208',	'F17209',	'F17210',	'F17211',	'F17213',
	'F17218',	'F17219',	'F17220',	'F17221',	'F17223',	'F17228',	'F17229',	'F17290',
	'F17291',	'F17293',	'F17298',	'F17299',	'T65292A',	'T65292S'		

© 2024 Williams FS et al. *JAMA Network Open.*

**Inhalant**								
	'F1810',	'F18120',	'F18121',	'F18129',	'F1814',	'F18150',	'F18151',	'F18159',
	'F1817',	'F18180',	'F18188',	'F1819',	'F1820',	'F1821',	'F18220',	'F18221',
	'F18229',	'F1824',	'F18250',	'F18251',	'F18259',	'F1827',	'F18280',	'F18288',
	'F1829',	'F1890',	'F18920',	'F18921',	'F18929',	'F1894',	'F18950',	'F18951',
	'F18959',	'F1897',	'F18980',	'F18988',	'F1899'			
**Other Psychoactive**								
	'F19130',	'F19131',	'F19132',	'F19139',	'F558',	'F1910',	'F19120',	'F19121',
	'F19122',	'F19129',	'F1914',	'F19150',	'F19151',	'F19159',	'F1916',	'F1917',
	'F19180',	'F19181',	'F19182',	'F19188',	'F1919',	'F1920',	'F1921',	'F19220',
	'F19221',	'F19222',	'F19229',	'F19230',	'F19231',	'F19232',	'F19239',	'F1924',
	'F19250',	'F19251',	'F19259',	'F1926',	'F1927',	'F19280',	'F19281',	'F19282',
	'F19288',	'F1929',	'F1990',	'F19920',	'F19921',	'F19922',	'F19929',	'F19930',
	'F19931',	'F19932',	'F19939',	'F1994',	'F19950',	'F19951',	'F19959',	'F1996',
	'F1997',	'F19980',	'F19981',	'F19982',	'F19988',	'F1999'		

© 2024 Williams FS et al. *JAMA Network Open.*

eFigure. Percentage of CMC Groups by Time Period

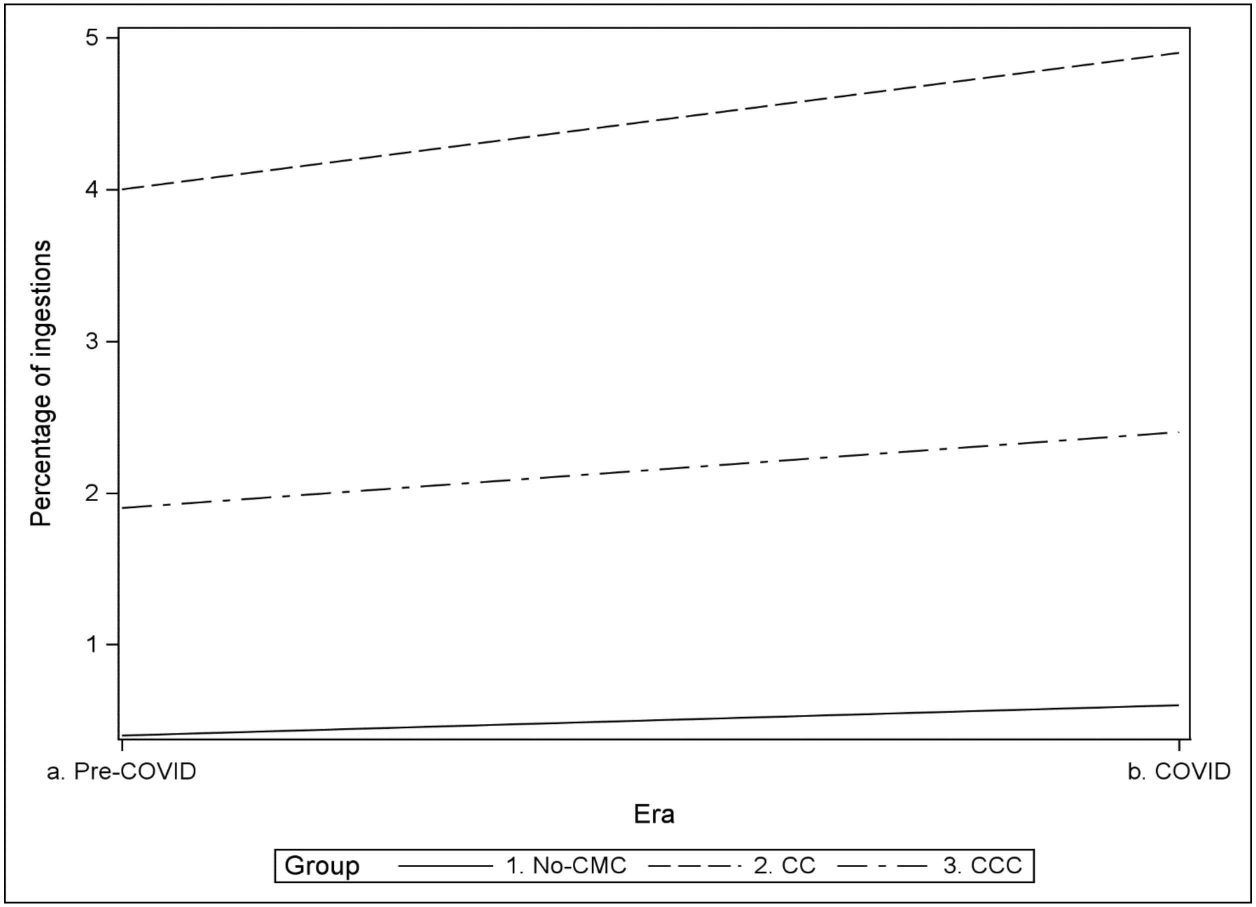

© 2024 Williams FS et al. *JAMA Network Open.*
