# Supplementary material for: Substance Use Emergency Department Visits Among Youths With Chronic Conditions During COVID-19
Source: JAMA Netw Open. 2024 Oct 4;7(10):e2435059. doi: 10.1001/jamanetworkopen.2024.35059 (PMC11581538; doi:10.1001/jamanetworkopen.2024.35059)
Supplement: Supplement 2. — Data Sharing Statement [file jamanetwopen-e2435059-s002.pdf]

Data Sharing Statement

Williams. Substance Use Emergency Department Visits Among Youths With Chronic Conditions During COVID-19. *JAMA Netw Open*. Published September 30, 2024. doi:10.1001/jamanetworkopen.2024.35059

Data
Data available: No
